# Impact of human monocyte and macrophage polarization on NLR expression and NLRP3 inflammasome activation

**DOI:** 10.1371/journal.pone.0175336

**Published:** 2017-04-12

**Authors:** Fawaz Awad, Eman Assrawi, Claire Jumeau, Sophie Georgin-Lavialle, Laetitia Cobret, Philippe Duquesnoy, William Piterboth, Lucie Thomas, Katia Stankovic-Stojanovic, Camille Louvrier, Irina Giurgea, Gilles Grateau, Serge Amselem, Sonia-Athina Karabina

**Affiliations:** 1 Sorbonne Université, UPMC Univ Paris 06, INSERM, UMR_S 933, Assistance Publique Hôpitaux de Paris, Hôpital Trousseau, Service de Génétique et d’Embryologie médicales, Paris, France; 2 Sorbonne Université, UPMC Univ Paris 06, INSERM, UMR_S 933, Assistance Publique Hôpitaux de Paris, Hôpital Tenon, Service de Médecine interne, Paris, France; Chang Gung University, TAIWAN

## Abstract

Inflammasomes are multiprotein complexes nucleating around an NLR (Nucleotide-binding domain and Leucine-rich Repeat containing protein), which regulate the secretion of the pro-inflammatory interleukin (IL)-1β and IL-18 cytokines. Monocytes and macrophages, the main cells expressing the inflammasome genes, adapt to their surrounding microenvironment by a phenotypic polarization towards a pro-inflammatory M1 phenotype that promotes inflammation or an anti-inflammatory M2 phenotype important for resolution of inflammation. Despite the importance of inflammasomes in health and disease, little is known about inflammasome gene expression in relevant human cells and the impact of monocyte and macrophage polarization in inflammasome gene expression. We examined the expression of several members of the NLR, caspase and cytokine family, and we studied the activation of the well-described NLRP3 inflammasome in an experimental model of polarized human primary monocytes and monocyte-derived macrophages (M1/M2 phenotypes) before and after activation with LPS, a well-characterized microbial pattern used in inflammasome activation studies. Our results show that the differentiation of monocytes to macrophages alters NLR expression. Polarization using IFN-γ (M1 phenotype), induces among the NLRs studied, only the expression of *NOD2*. One of the key results of our study is that the induction of *NLRP3* expression by LPS is inhibited in the presence of IL-4+IL-13 (M2 phenotype) at both mRNA and protein level in monocytes and macrophages. Unlike caspase-3, the expression of inflammasome-related *CASP1* (encodes caspase-1) and *CASP4* (encodes caspase-4) is up-regulated in M1 but not in M2 cells. Interestingly, the presence of LPS marginally influenced *IL18* mRNA expression and secretion, unlike its impact on *IL1B*. Our data provide the basis for a better understanding of the role of different inflammasomes within a given environment (M1 and M2) in human cells and their impact in the pathophysiology of several important inflammatory disorders.

## Introduction

Inflammation is characterized by increased cytokine secretion and activation of innate immunity cells. In response to stimuli present in their local microenvironment or in circulation, monocytes and macrophages modulate their gene expression towards either a pro-inflammatory M1 or an anti-inflammatory M2 phenotype. M1 cells play an important role in host defense through cytokine production, whereas M2 cells mediate tissue repair and homeostasis [1–6]. This adaptation of monocytes and macrophages to their immediate microenvironment is known as polarization and can occur at any time during inflammation [7]. However, the impact of the polarization status in human pathophysiology and the related inflammatory signaling remains poorly understood.

Innate response during inflammation is assured by the family of the evolutionarily conserved NLR receptors (Nucleotide-binding domain and Leucine-rich Repeat containing proteins) expressed mainly in monocytes and macrophages [8]. Upon detection of microbial (Pathogen-associated molecular patterns, PAMPs) or cell damage molecular patterns (Damage-associated molecular pattern, DAMPs), NLRs initiate the formation of multiprotein intracellular complexes called inflammasomes, which regulate secretion of the bioactive forms of the IL-1 cytokine family, through association with the adaptor protein ASC (Apoptosis-associated Speck-like protein containing a Caspase recruitment domain), and activation of caspase-1 (encoded by *CASP1*) [9–11]. The secreted IL-1β and IL-18 play key roles in systemic inflammation promoting the expression of pro-inflammatory genes and thereby amplifying the inflammatory response. NLRs are multi-domain proteins. Their C-terminal domain is rich in leucine repeats (LRR), which under resting conditions, auto-inhibits the NLR [12,13]. The central nucleotide-binding domain (NACHT), mediates oligomerization and a variable N-terminal effector domain subdivides the NLR family into subgroups (S1 Fig) [14]. Among NLRs, NLRP1 [15], NLRP2 [16], NLRP3 [16], NLRP6 [17], NLRP7 [18], NLRP12 [19], NLRC4 and NLRB/NAIP [20] are considered as inflammasome-nucleating proteins. The NLRP3 inflammasome gained a lot of attention due to the causality of *NLRP3* mutations in cryopyrin-associated periodic syndromes (CAPS) [21,22], an auto-inflammatory pathology characterized by high levels of IL-1β secretion from peripheral blood mononuclear cells (PBMCs) of patients.

Inflammasomes are key regulators of pro-inflammatory cytokine secretion, and despite their importance in several diseases and in host defense [10,11,23,24], the expression profile of the assembling NLRs and inflammasome-related genes in human immune cells is still elusive. Although mice have provided important insight into the understanding of the pathophysiology of human diseases, important differences exist between species including differences in innate immunity cells and sensitivity to LPS [25–27]. We undertook this study to examine the links between the polarization status of monocytes and macrophages and the expression of key molecules involved in inflammasome formation and activation in human-relevant primary cells (PBMCs, M1 and M2 monocytes and macrophages). These data should open new lines of research for a better understanding of the role of different inflammasomes at a given environment (M1 and M2) and their impact in the pathophysiology of several important inflammatory diseases.

## Materials and methods

### Peripheral blood mononuclear cell, monocyte and macrophage isolation

Peripheral blood mononuclear cells were isolated from buffy coats provided by the “*Etablissement Francais du Sang*, *(EFS)*” from apparently healthy blood donors, using Ficoll density gradient as described previously [28]. Monocytes were selected by 1h adherence and cultured for 24h in the presence of RPMI 1640 complemented with 10% pooled human serum (HS), penicillin and streptomycin (complete medium). Monocyte-derived macrophages were obtained after 7 days culture of monocytes in the presence of complete medium (named macrophages from here after).

### Monocyte and macrophage polarization

Monocytes after adherence or macrophages (after 7 days in culture) were treated either with 100 ng/ml interferon (IFN)-γ (to polarize towards an M1 phenotype) or with 10 ng/ml IL-4+IL-13 (to polarize towards an M2 phenotype) or with complete medium alone (control cells, M0) for 24h. The last 3h, cells were treated with 100 ng/ml LPS. Following treatments cell lysates were collected for RNA and protein expression and cell culture supernatants were used for ELISA.

### Reverse Transcription and quantitative-PCR (RT-qPCR)

Total RNA from PBMCs, monocytes or macrophages, treated or not, was isolated using the RNAeasy mini kit (Qiagen) including a DNase step (Qiagen). RNA was reversed transcribed in the presence of 2.5 mM oligo-dT using the Transcriptor High Fidelity cDNA Synthesis Kit (Roche), following the manufacturer’s instructions. 5 ng of cDNA was amplified using the Mesa Blue qPCR MasterMix Plus for SYBR Assay (Eurogentec) in the Light Cycler LC480 (Roche). mRNA expression was normalized to the levels of ribosomal protein L13a, *RPL13A* (NM_012423.2), which was used as housekeeping gene. The relative level of expression of a gene between sample 1 (treated) and sample 2 (control) was calculated using the ΔΔCt formula: 2^−(Ct1−Ct *RPL13A* 1)−(Ct2−Ct *RPL13A* 2)^. Normalized Ct values (mean of controls) were used to calculate the gene expression ratio between two samples. Primers (S1 Table) were designed using the Probe Finder software (http://qpcr.probefinder.com/organism.jsp; Roche Life sciences)

### NLRP3 inflammasome activation

Polarized monocytes and macrophages were treated with LPS 100 ng/ml for 3, 7 or 24 h. In order to induce NLRP3 inflammasome activation, cells were additionally stimulated with 5 mM of adenosine triphosphate (ATP) (Sigma Aldrich) for the last 30 minutes. Supernatants were collected and stored at -20°C for cytokine measurement by ELISA.

### Western blot analysis

PBMCs, monocytes or macrophages were lysed in (5×) Laemmli SDS buffer supplemented with 1% β-mercaptoethanol, 0.01% bromophenol blue and protease inhibitor cocktail (Roche). Equal amounts of protein were analyzed by SDS-PAGE. Proteins were transferred to PVDF membranes and incubated with primary and secondary antibodies following the manufacturers’ instructions. Primary antibodies against ASC (1:2000, AL177), caspase-1 (1:1000, Bally-1) and NLRP3 (1:1000, Cryo-2) were from Adipogen. Anti-IL-1β (1:500, AF201) antibody was from R&D. Anti-GAPDH and anti-Actin antibodies (1:5000) were from Cell Signaling. Secondary HRP-linked antibodies against rabbit and mouse IgG (1:2000) were from Cell Signaling and against goat IgG (1:2000) was from R&D. Immunoblots were revealed using the enhanced chemiluminescence reagent (Thermo Scientific) and visualized using a BIORAD camera (Universal Hood II). GAPDH or Actin was used as loading controls.

### ELISA

Cell culture supernatants from human monocytes and macrophages were collected after treatments, centrifuged for 10 minutes at 250×g and kept at -20°C for cytokine measurements. IL-1α, IL-1β, IL-1ra, and TNF-α were quantified using the DuoSet ELISA kits (R&D). For IL18, we used the human IL18 ELISA kit (MBL). All measurements were made following manufacturers’ instructions.

### Statistics

Results are presented as the mean ± standard error of the mean (SEM) from experiments performed in duplicate in cells from at least 4 independent donors. The Mann Whitney was used to evaluate the statistical significance of differences between 2 groups. A value of P<0.05 was considered significant. GraphPad Prism software was used for data organization, analysis and graphing.

## Results

### Expression of NLRs, caspases and cytokines in human PBMCs, monocytes and macrophages

**Fig 1:**
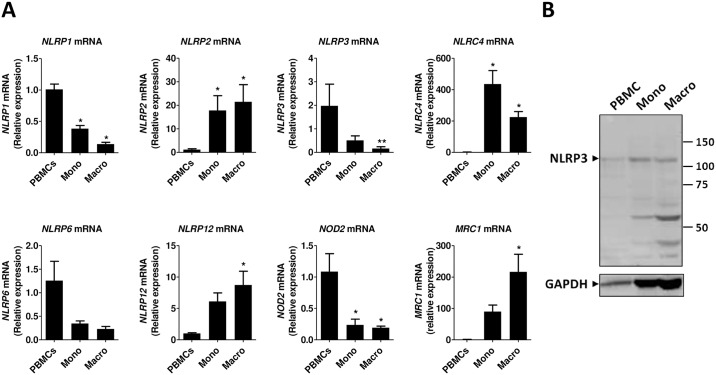
*NLR* expression in human PBMCs, monocytes and macrophages. (A) mRNA gene expression was measured by RT-qPCR and presented as relative fold change of PBMCs. Data represent the mean ± standard error of the mean (SEM) of ≥ 4 experiments performed in duplicate in cells isolated from ≥ 4 independent donors. Mono: monocytes; Macro: macrophages. Asterisks indicate significant differences as compared to PBMCs (Mann Whitney test: * p < 0.05; ** p < 0.01). (B) Western blot analysis in total lysates of human PBMCs, monocytes and macrophages using anti-NLRP3 antibody. GAPDH was used as loading control. The blot shown is representative of at least 5 experiments from independent donors. Mono: monocytes; Macro: macrophages.

**Fig 2:**
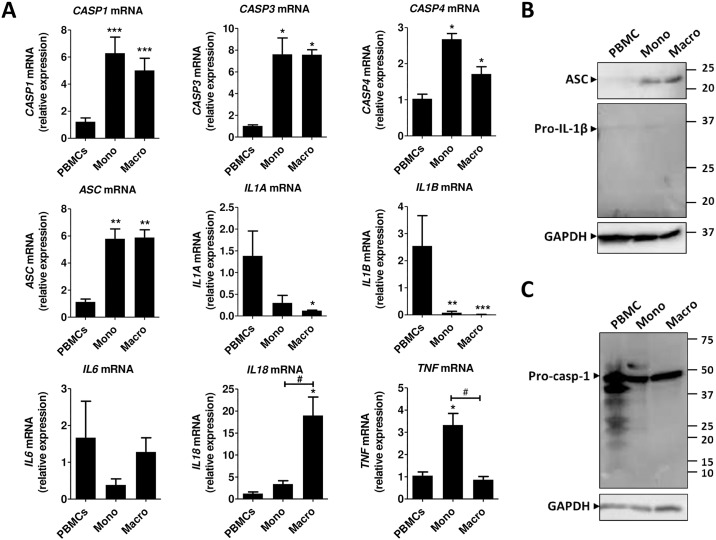
*CASP* and cytokine expression in human PBMCs, monocytes and macrophages. (A) mRNA gene expression was measured by RT-qPCR and expressed as relative fold change of PBMCs. Data represent the mean ± SEM of ≥ 4 experiments performed in duplicate in cells isolated from ≥ 4 independent donors. Asterisks indicate significant differences as compared to PBMCs (Mann Whitney test: * p < 0.05; ** p < 0.01; *** p < 0.001); (#) indicates significant differences between monocytes and macrophages (Mann Whitney test: # p < 0.05). (B&C) Western blot analysis in total lysates from human PBMCs, monocytes and macrophages using an anti-ASC, an anti-caspase-1 or an anti-IL-1β antibody. GAPDH was used as loading control. Western blots are representative of at least 5 independent experiments. Mono: monocytes; Macro: macrophages; casp-1: caspase-1.

We initially studied the mRNA expression of different NLR genes in human PBMC, monocytes and macrophages. As shown in Fig 1A, the expression of *NLRP1*, *NLRP3*, *NLRP6* and *NOD2*, which is already low in PBMC (Ct >28), is further decreased during differentiation of adherent monocytes to macrophages. The opposite happens with *NLRP2* and *NLRC4* and *NLRP12* whose expression is induced from PBMCs to monocytes and macrophages (Fig 1A). The expression of *MRC1*, a marker of monocyte differentiation to macrophages, is accordingly induced. The gene expression of *CASP1*, *CASP3*, *CASP4* and *ASC* is increased in monocytes and macrophages as compared to PBMCs, but does not change significantly between monocytes and macrophages (Fig 2A). The mRNA expression of *IL1A* and *IL1B* decreases from PBMCs to monocytes and to macrophages, whereas *IL18* expression is low in PBMCs and monocytes (Ct >30) but increases in macrophages. *IL6* mRNA expression does not change significantly between PBMCs, monocytes and macrophages, whereas the expression *TNF* is induced in monocytes as compared to PBMCs and macrophages (Fig 2A).

We then tested the protein expression of NLRP3 inflammasome components in PBMCs, monocytes and macrophages. NLRP3 protein expression was found to vary among donors but detectable most times in PBMCs, monocytes and macrophages. One major band was detected around 120 kDa, corresponding to the expected molecular weight, and some strong potentially non-specific bands appeared in lower molecular weights (Fig 1B and data not shown). ASC protein expression followed the gene expression pattern and was found to be up-regulated in monocytes and macrophages as compared to PBMCs (Fig 2B). Several bands were detected in PBMCs including pro-caspase-1, the zymogen form of caspase-1, and a fragment at about 20 KDa, which correspond to the p20 active form of caspase-1. Mainly the pro-caspase-1 was detected in monocytes and macrophages (Fig 2C). As expected, without stimulation, pro-IL-1β the inactive form of IL-1β, was hardly detectable in PBMCs, monocytes and macrophages (Fig 2B).

### Expression of NLRs, caspases and cytokines in human monocytes polarized towards a M1 or M2 phenotype in the presence or absence of LPS

**Fig 3:**
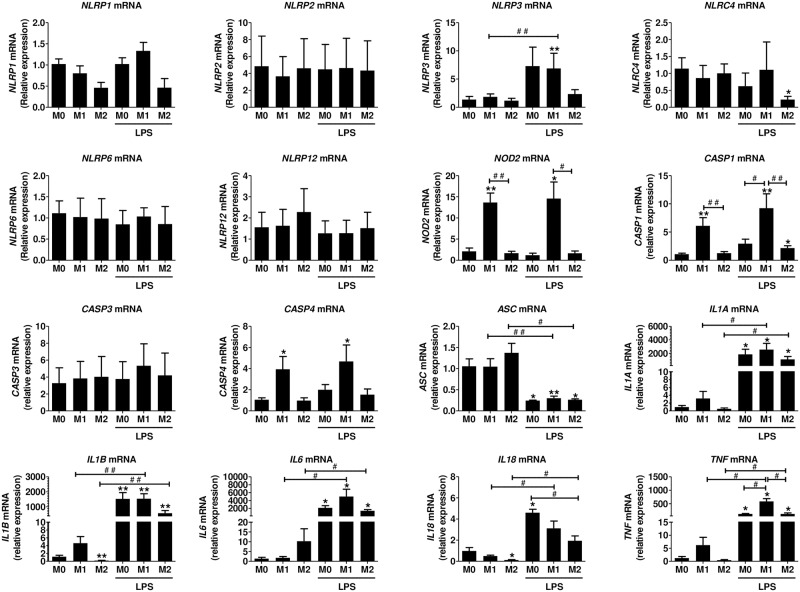
NLR, *CASP* and *cytokine* gene expression in human polarized monocytes. Monocytes were polarized towards M1 or M2 and stimulated with 100 ng/ml LPS for 3h as described in the methods. mRNA was isolated and gene expression was measured by RT-qPCR and expressed as relative fold change of M0. Data represent the mean ± SEM of ≥ 4 experiments performed in duplicates in cells isolated from ≥ 4 independent donors. M0: cells treated with complete medium (control); M1: cells treated with 100 ng/ml IFN-γ (polarized towards M1); M2: cells treated with 10 ng/ml IL-4+IL-13 (polarized towards M2). Asterisks indicate significant differences as compared to M0 (Mann Whitney test: * p < 0.05; ** p < 0.01); (#) points out significant differences between the indicated groups (Mann Whitney test: # p < 0.05; ## p < 0.01).

We next studied the gene expression of NLRs in polarized monocytes in the presence or absence of LPS, a common stimulus used for inflammasome activation. Polarization markers *CXCL10* for M1 and *ALOX15* for M2 were significantly up-regulated in the respective phenotypes (S2 Fig -upper panel). The expression of *NLRP1* was down-regulated in M2 monocytes in the presence or absence of LPS (Fig 3), but this result did not reach statistical significance, as the expression levels of *NLRP1* were already low in M0 monocytes. Expression of *NLRP2* and *NLRP6* was low (Ct >30) and was not affected by polarization or LPS treatment (Fig 3). *NLRP3* expression was low in M0, M1 and M2 monocytes. Most importantly, LPS significantly induced *NLRP3* expression in M0 and M1 cells, but in M2 monocytes, there was either lack or an inhibition of the induction. *NLRC4* expression was not modulated by polarization but in the presence of LPS in the M2 cells, the expression was significantly decreased. *NLRP12* was slightly increased in the M2 monocytes as compared to M0 or M1 cells and was not significantly modulated by LPS. *NOD2* expression was strongly up-regulated in M1 cells and this result was not dependent on the presence or absence of LPS. In summary, the expression of NLRs is low in M0 human adherent monocytes. Among the NLRs tested, only the gene expression of *NOD2* was significantly induced in M1 cells, whereas LPS differentially modulated the expression of *NLRP3* and *NLRC4* between M1 and M2 cells.

Inflammasome-related *CASP1* and *CASP4* mRNA expression was strongly up-regulated in M1 monocytes and in the presence of LPS, this result was more pronounced (Fig 3). Effector *CASP3* gene expression was not modulated by monocyte polarization. *ASC* expression was not modulated by monocyte polarization, but in the presence of LPS, there was a significant decrease in all conditions (M0, M1, and M2). mRNA expression of *IL1A* and *IL1B* was slightly up-regulated in the presence of INF-γ (M1 cells) (Ct ≈ 27 and Ct ≈33 respectively), and significantly increased (more that 1000-fold) in the presence of LPS. In M0 and M1 cells, the effect of LPS was stronger as compared to M2 monocytes. *IL6* mRNA was significantly increased (more than 2000-fold) in the presence of LPS and this effect was stronger in M1 cells (Fig 3). *IL18* mRNA was low in M0 (Ct <30) and M1 (Ct <31) monocytes, as compared to *IL1B* and significantly lower in M2 cells (Ct < 32). In the presence of LPS, a small 2 to 3-fold increase in *IL18* mRNA was observed in M0 and M1 cells; the increase was less important in M2 monocytes. *TNF* mRNA was up-regulated in the presence of IFN-γ. In the presence of LPS, the expression was increased more than 200 times in all conditions and this effect was more obvious in M1 monocytes.

### NLRP3 inflammasome protein expression in polarized monocytes

**Fig 4:**
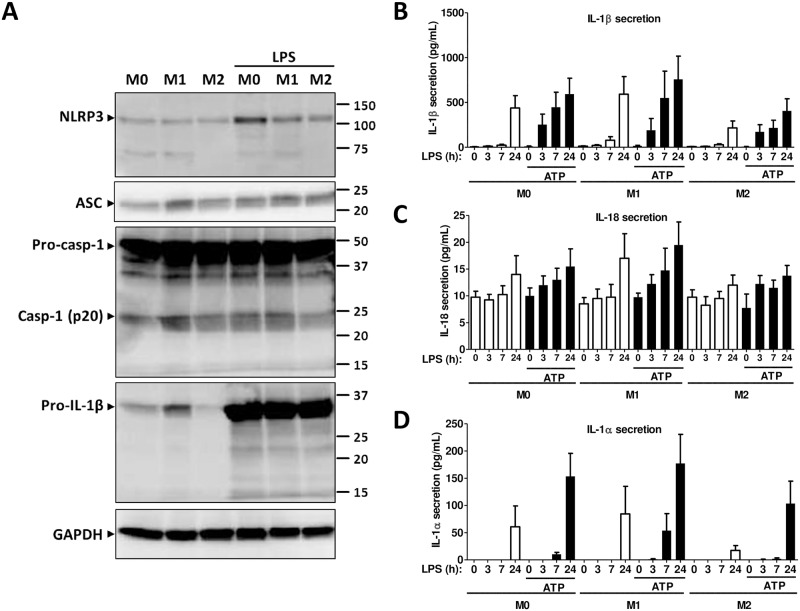
NLRP3 inflammasome protein expression in polarized monocytes. (A) Western blot analysis from total cell lysates of M0, M1 or M2 monocytes in the presence or absence of LPS using an anti-NLRP3, an anti-ASC, an anti-caspase-1 or an anti-IL-1β antibody. GAPDH was used as loading control. Blots are representative of ≥ 3 independent experiments. (B-D) IL-1β, IL-1α and IL-18 secretion as assessed by ELISA in cell culture supernatants of M0, M1, or M2 monocytes after activation of NLRP3 inflammasome with 100 ng/ml LPS for the indicated time in the presence or absence of 5mM ATP for the last 30 minutes. M0: cells treated with complete medium (control); M1: cells treated with 100 ng/ml IFN-γ (polarized towards M1); M2: cells treated with 10 ng/ml IL-4+IL-13 (polarized towards M2). Data represent the mean ± SEM of ≥ 4 independent experiments done in monocytes isolated from buffy coats of ≥ 4 independent donors.

Similar to gene expression, NLRP3 protein expression was significantly up-regulated in the presence of LPS in M0 or M1 monocytes, whereas in M2 monocytes, there was lack or inhibition of NLRP3 induction (Fig 4A), underscoring the importance of the local microenvironment on NLRP3 expression. ASC protein expression was detectable in M0, M1 or M2 monocytes and was not significantly modulated by LPS (Fig 4A). Protein expression of pro-caspase-1 (45 kDa) was very strong under all conditions. A band corresponding to the molecular weight of the cleaved caspase-1 (p20) was apparent in all conditions but the intensity of the band was weaker in the M2 cells in the presence or absence of LPS (Fig 4A). Following the gene expression pattern, pro-IL-1β was strongly expressed in M1 but was almost absent in M2 monocytes. Treatment with LPS strongly induced the protein expression in all conditions. Several low molecular bands, including one corresponding to the molecular weight of the active form (17 kDa), was present in M0, M1 or M2 monocytes after treatment with LPS (Fig 4A) suggesting inflammasome activation.

### Cytokine secretion during NLRP3-dependent inflammasome activation in human polarized monocytes

We then tested cytokine secretion during NLRP3 inflammasome activation in M0, M1 or M2 cells using time-dependent-LPS priming and exogenous ATP during the last 30 minutes [29–31]. Inflammasome-dependent IL-1β secretion started after a 3h treatment with LPS and ATP, whereas in the presence of LPS alone, IL-1β secretion was detected after a 24h treatment, indicating the importance of the two signals for robust activation of inflammasome in human monocytes (Fig 4B). These results were similar in M0, M1 and M2 cells; however, in M2 monocytes, IL-1β secretion was lower at all-time points. Interestingly, a 24h treatment with LPS only slightly increased IL-18 secretion, which was generally very low in M0, M1 or M2 cells (Fig 4C). Addition of ATP had a marginal if any impact in M0 or M1 monocytes. In M2 cells, the secretion was very low at all-time points (Fig 4C). IL-1α, which can be secreted by caspase-1- dependent and independent pathways [32,33], was secreted only after 24h incubation with LPS, whereas the secretion started at 7h in the presence of ATP in M1 but not in M0 or M2 cells (Fig 4D). Neither time-dependent exposure to LPS nor ATP had an impact on IL-1ra secretion, which remained higher in M2 monocytes as compared to M1 or to M0 cells (S3 Fig). TNF-α secretion, which is not modulated by the inflammasome, started after 3h incubation with LPS, whereas addition of ATP as expected had no additional impact. TNF-α secretion remained lower in M2 monocytes as compared to M0 or M1 in all conditions (S3 Fig). These results indicate that the local microenvironment impacts the activation of the inflammasome and thus the regulation of IL-1β secretion. The secretion profile of inflammasome-regulated IL-18 and the kinetics of IL-1α secretion are of particular interest and potentially highlight the different roles these cytokines may play as alarmins under different inflammatory environments.

### Expression of NLRs, caspases and cytokines in human macrophages polarized towards a M1 or M2 phenotype in the presence or absence of LPS

**Fig 5:**
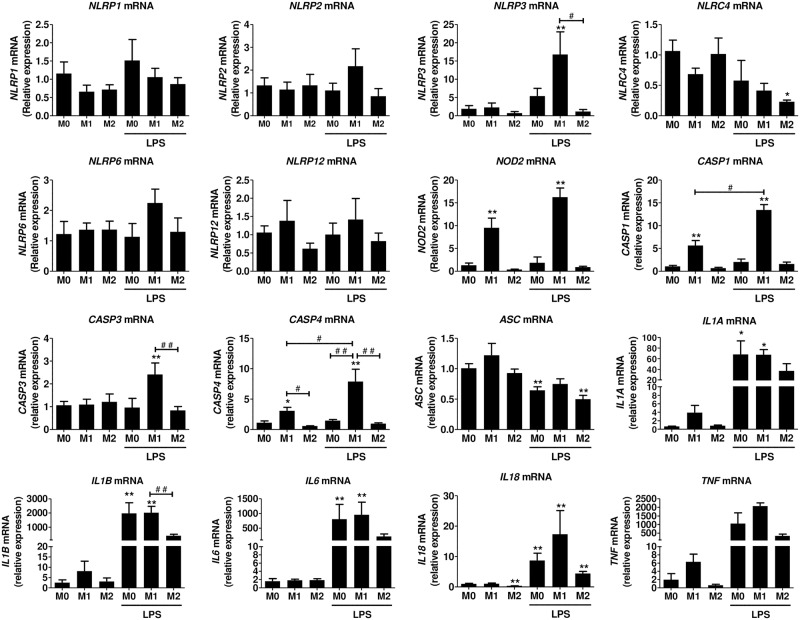
NLR, CASP and cytokine gene expression in human polarized macrophages. Monocyte derived macrophages were polarized towards M1, M2, or M0 and stimulated with 100 ng/ml LPS for 3h as described in the methods. mRNA was isolated and gene expression was measured by RT-qPCR and expressed as relative fold change of M0. Data represent the mean ± SEM of ≥ 4 experiments performed in duplicates in cells isolated from independent donors. M0: cells treated with complete medium (control); M1: cells treated with 100 ng/ml IFN-γ (polarized towards M1); M2: cells treated with 10 ng/ml IL-4+IL-13 (polarized towards M2). Asterisks indicate significant differences as compared to M0 (Mann Whitney test: * p < 0.05, ** p < 0.01); (#) points out significant differences between the indicated groups (Mann Whitney test: # p < 0.05; ## p < 0.01).

Polarization markers, *CXCL10* for M1 and *ALOX15* for M2, were significantly up-regulated in the respective phenotypes as shown in S2 Fig -lower panel. The expression of *NLRP1*, *NLRP2* and *NLRP6* and *NLRP12* was low (Ct >30) and was not significantly modulated during macrophage polarization in the presence or absence of LPS. *NLRP3* was up-regulated in the presence of LPS and interestingly, this effect was stronger in M1 macrophages, whereas there was lack or inhibition of induction in M2 macrophages, similar to what we saw in monocytes. *NLRC4* (Ct <28 before LPS treatment) expression was down-regulated by LPS (Ct <29 after LPS treatment), an effect seen mainly in M2 macrophages. *NOD2* was significantly up-regulated in M1 macrophages in the presence or absence of LPS. Similarly to what we observed in monocytes, the expression of inflammasome-related *CASP1* and *CASP4* was significantly up-regulated in M1 macrophages and treatment with LPS further induced this effect. *CASP3* mRNA was induced 2-fold in M1 macrophages treated with LPS. *ASC* mRNA expression was not affected by the polarization status and a minor decrease was seen in the presence of LPS (Fig 5).

The polarization status only slightly induced the mRNA expression of *IL1B*, *IL6* and *TNF*. In the presence of LPS, their mRNA levels were increased more than a thousand-fold in M0 and M1 macrophages. *IL18* and *IL1A* mRNA levels were also significantly induced by LPS, but to a much lesser extent than what observed with *IL1B*, *IL6*, *TNF* (Fig 5). In M2 cells, in the presence of LPS, there was an inhibition of the cytokine gene expression (Fig 5).

### NLRP3 inflammasome protein expression in polarized macrophages

**Fig 6:**
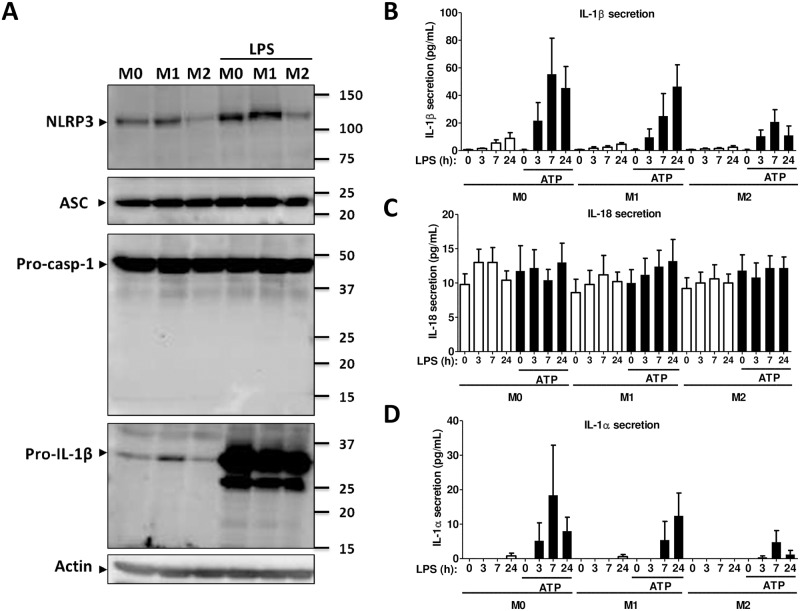
NLRP3 inflammasome protein expression in polarized macrophages. (A) Western blot analysis from total cell lysates of M0, M1 or M2 macrophages in the presence or absence of LPS using an anti-NLRP3, an anti-ASC, an anti-caspase-1 or an anti-IL-1β antibody. GAPDH was used as loading control. Blots are representative of ≥ 3 independent experiments. (B-D) IL-1β, IL-1α and IL-18 secretion as assessed by ELISA in cell culture supernatants of M0, M1, or M2 monocytes after activation of NLRP3 inflammasome with 100 ng/ml LPS for the indicated period of times in the presence or absence of 5mM ATP for the last 30 minutes. M0: cells treated with complete medium (control); M1: cells treated with 100 ng/ml IFN-γ (polarized towards M1); M2: cells treated with 10 ng/ml IL-4+IL-13 (polarized towards M2). Data represent the mean ± SEM of ≥ 4 independent experiments done in macrophages derived from monocytes of ≥ 4 independent donors.

NLRP3 protein expression in macrophages followed the mRNA expression and was up-regulated in the presence of LPS in M0 or M1 macrophages but not in M2 (Fig 6A). In the presence of LPS, ASC protein expression was not significantly modulated. The zymogen form of pro-caspase-1 (45 kDa) was present in all conditions, whereas the cleaved form was not detected (Fig 6A). This result is in line with the hypothesis that macrophages need two signals for inflammasome activation: LPS, which induces the mRNA expression of *IL1B* and *NLRP3*, and a second signal which induces the autoproteolytic cleavage of pro-caspase-1 [32]. Similar to caspase-1, only the pro-form of IL-1β was detected in LPS-treated macrophages, although several fragmented forms were present probably due to the very strong expression of the zymogen form. In the absence of LPS, pro-IL-1β expression was lower in M2 macrophages (Fig 6A).

### Cytokine secretion in human polarized macrophages during NLRP3-dependent inflammasome activation

LPS alone induced a low IL-1β secretion after 7h incubation in M0, M1 and M2 cells, whereas in the presence of ATP, the secretion of IL-1β was higher and started after 3h. In M2 macrophages, the IL-1β secretion was lower than in M1 or M0 cells (Fig 6B). IL-18 secretion was very low and was not increased by treatment with ATP (Fig 6C). IL-1α secretion increased only after 24h incubation with LPS in M0, M1 or M2 cells, whereas the presence of ATP induced a small secretion of IL-1α in M0 and M1 cells (Fig 6D). IL-1ra was not affected by treatment with LPS or ATP, and secretion levels were higher in M2 macrophages (S4 Fig). LPS induced TNF-α secretion in a time-dependent manner, whereas ATP had no effect. TNF-α secretion was lower in M2 cells (S4 Fig).

## Discussion

Although the importance of inflammasomes in host defense and in many inflammatory and metabolic diseases is established [10,11,23,24], the majority of the experimental data has come from work in mice, or from overexpression systems in human cell lines that are not relevant to innate immunity [33–36]. In the current study, we used human primary monocytes and monocyte-derived macrophages to study inflammasome gene expression. Despite being intracellular complexes, inflammasomes regulate secretion of the IL-1 family of pro-inflammatory cytokines, thereby defining the immediate environment of the host cell. Monocytes and macrophages adapt to their surrounding conditions by phenotypic polarization towards pro-inflammatory M1 or anti-inflammatory M2 phenotype that display opposing activities. M1 cells promote inflammation, whereas M2 cells are critical for inflammation resolution. Here, we provide data on the expression of several NLR family members, caspases and cytokines in human polarized monocytes and monocyte-derived macrophages (M1/M2 phenotypes) before and after activation by LPS [16,32].

The plasticity of macrophages has been described *in vivo* in different pathophysiological conditions [37]. The polarization phenotype indicate that under specific conditions in their microenvironment, pro- or anti- inflammatory responses (associated with M1 & M2 phenotypes, respectively) dominate at any given time point. In other words, genes tested in M1 and M2 polarized cells will be expressed in both types of cells. But a specific stimulus (e.g., pathogen) will commonly up-regulate sets of anti-inflammatory genes in M2 cells, which are “primed” for resolution of inflammation, whereas in M1 “primed” cells, the same set of genes will be down-regulated. We tested whether or not polarization of human monocytes and macrophages impacts activation of inflammasome in the most relevant *in vitro* environment. Under basal conditions in monocytes and macrophages, NLR expression is low, whereas a pro-inflammatory environment (*i*.*e*., IFN-γ treatment leading to M1 phenotype) induces only the expression of *NOD2*, suggesting that increased IFN-γ levels in the local environment is not a triggering factor for all NLRs.

Microbial patterns (PAMPs) are sensed by specific NLRs. The effect of LPS induced specifically the expression of *NLRP3*, in line with previously reported results in human monocytes and macrophages [38,39] and is potentially NF-κB-dependent [40]. However, in M2 cells (*i*.*e*., polarization with IL-4, IL13), the effect of LPS in inducing *NLRP3* expression was inhibited and this is one of the important results of this study. Although many different PAMPs and DAMPs activate NLRP3 inflammasome, the molecular mechanisms governing *NLRP3* expression and activation still remain puzzling. The impact of LPS on *NLRP3* expression can be either at the transcriptional level, or, during shorter incubation periods, LPS can induce NLRP3 deubiquitination leading to inflammasome activation [41]. In our system, the effect of LPS in M1 cells seems to be at the transcriptional level, whereas in M2 cells, a potential impact of LPS on NLRP3 ubiquitination cannot be excluded and is worth further investigation. Very few molecules are known to down-regulate *NLRP3* expression at both mRNA and protein levels [42,43]. In the murine *Nlrp3* promoter, aryl hydrocarbon receptor binding sites have been identified suggesting that several exogenous and endogenous ligands can act as repressors of *Nlrp3* transcription [42]. Tight regulation of *NLRP3* is critical for inflammatory outcome, and anti-inflammatory conditions (M2 phenotype) assure that the NLRP3 inflammasome is not activated. One would therefore expect that an M1 monocyte/macrophage phenotype prevails in inflammatory diseases associated with *NLRP3* mutations, but this remains to be addressed.

Although basal levels of *CASP1*, *CASP3* and *CASP4* are present in PBMCs, their expression was found to be up-regulated in monocytes and macrophages. Interestingly, IFN-γ (M1 stimulus) specifically induced the mRNA of the inflammasome-assembling caspases *CASP1* and *CASP4* but not *CASP3*. Caspase 1 is considered as a canonical inflammasome partner, whereas caspase 4 is mainly described as part of a non-canonical inflammasome as it can directly bind intracellular LPS and regulate cytokine secretion [44]. These results correlated with an increased presence of the active form of caspase-1 in lysates of M1 monocytes. Additionally, in M1 monocytes, pro-inflammatory cytokine expression was induced. The NLRP3 inflammasome is commonly activated after receiving a priming signal from LPS through TLR4 and a second signal from a variety of PAMPs or DAMPs including from purinergic receptors in response to ATP [45,46]. The effect of NF-κB-dependent LPS modulation of cytokine mRNA expression was important in all cytokines apart from *IL18*. Inflammasome-dependent IL-1β secretion in monocytes was seen only after 24h incubation with LPS, whereas IL-1α and IL-18 secretion was very modest even after lengthy incubations. The presence of ATP, accelerated the kinetics of IL-1β and IL-1α and marginally IL-18 secretion, but had no effect on TNF-α or IL-1ra, which do not depend on the inflammasome.

M0, M1 or M2 macrophages, like monocytes, showed a similar regulation of NLR, caspase and cytokine mRNA expression, which suggests that monocytes, the first cells to encounter PAMPs and/or DAMPs, rapidly adapt to the environmental conditions by regulating the expression of inflammasome genes. These “acquired characteristics” are retained during monocyte migration to tissues and differentiation into resident macrophages. However, in macrophages, we were not able to detect the active form of the caspase-1, regardless of the polarization status of the cells. This result is in accordance with secreted IL-1β levels in macrophages which, even after lengthy treatments with LPS were almost 10 times lower as compared to monocytes. Although ATP induced IL-1β secretion in M0, M1 and to a lesser extend in M2 macrophages, the secretion levels were still very low compared to those seen in monocytes. IL-18 was marginally influenced by the presence of LPS at both mRNA and secreted protein levels. ATP had no additional effect on IL-18 secretion in macrophages. IL-18 is considered as an inflammasome-regulated cytokine [10] but our results suggest that its modulation is not similar to that of IL-1β, and additional pathways may regulate its levels. In contrast, IL-1α regulation in M1 or M2 macrophages is very similar to that of IL-1β in the conditions tested. In agreement with recent studies [47–49], this result supports the idea that IL-1α levels may also be regulated through inflammasome. Whether IL-1α secretion is a caspase-1 or caspase-4 -dependent inflammasome worth further studies. Its secretion should thus be measured in plasma or cell supernatants in order to better understand its role in inflammatory diseases.

In our study, secretion of all tested cytokines was lower in M2 as compared to M1 monocytes apart from IL-1ra, an anti-inflammatory cytokine [50] whose levels were constantly higher in M2 cells in all conditions tested. These results are in agreement with a previous study showing that the effect of E.coli and ion channel receptor P2X7R on NLRP3 inflammasome activation is different in M1 as compared to M2 macrophages [51]. In addition, the authors showed that release of IL-1β may be an extracellular stimulus for monocyte and macrophage polarization.

In our study, we also compared expression levels among PBMCs, human monocytes and monocyte-derived macrophages. We used PBMCs because these cells are easily accessible from patients’ blood and are commonly used in inflammasome-related studies (e.g., [35,52–54]). Monocytes were then enriched from PBMCs, through an adherence step. We observed that, for some NLRs, the mRNA expression levels were higher in PBMCs than in monocytes. Several hypotheses can explain this observation. First of all, the basal levels of the majority of the NLRs are low (28–35 Ct). As PBMCs are a heterogeneous population, which contain cells from myeloid and lymphoid origin, one cannot exclude that, during the isolation of PBMCs from total blood, a few polymorphonuclear cells may persist, which also express inflammasome genes [55]. Subtle differences due to the presence of polymorphonuclear cells may impact the NLR expression and explain the differences seen between PBMCs and monocytes (*i*.*e*., *NLRP1*, *NOD2)*. Secondly, we observed that NLR expression is modulated by adherence (from PBMCs to monocytes) and by the differentiation of monocytes into macrophages. The fact that the adherence, which implies cytoskeleton changes, impacts NLR expression is not surprising, as several studies showed that mechanistic links exist between NLRs and cytoskeleton [56].

The simultaneous expression of different NLRs in the same cell suggests that interactions among NLRs may exist at the protein level. Such interactions are possible through homotypic domain interactions [57]. In that line, *Salmonella* infection has recently been shown to induce recruitment of both NLRP3 and NLRC4 to the same inflammasome complex along with ASC, caspase-1, and caspase-8 [58,59]. NLR expression is not redundant and depends on the differentiation state of the mononuclear cells. It would be thus interesting to understand the role of each NLR in different immune cell types in basal or disease conditions.

In conclusion, this study provides extensive data on gene and protein expression of several NLR and NLRP3 inflammasome-related components under pro- or anti-inflammatory conditions in relevant human primary cells. Understanding inflammasome gene expression and regulation will help better take in charge inflammatory conditions. Recent work has shown that elevated and persistent expression of inflammasome genes in older individuals is associated with all causes of mortality [60]. Our results underline that at a given time and under a given stimulus, cells modulate their expression to adapt to the environment. The data obtained clearly show that *NLRP3* expression is negatively regulated under M2 conditions, a result that can open new avenues of research and new ways of regulating the activation of the NLRP3 inflammasome.

## Supporting information

S1 Fig Schematic representation of the domain structure of NLR proteins. (PDF)

S2 Fig Gene expression of polarization markers in monocytes and macrophages. Monocytes and macrophages were polarized towards M1, M2, and stimulated with 100 ng/ml LPS for 3h as described in the methods. mRNA was isolated and gene expression was measured by RT-qPCR and expressed as relative fold change of M0. Data represent the mean ± SEM of ≥ 4 experiments performed in duplicates in cells isolated from ≥ 4 independent donors. M0: cells treated with complete medium (control); M1: cells treated with IFN-γ (polarized towards M1); M2: cells treated with IL-4+IL-13 (polarized towards M2). Asterisks indicate significant differences as compared to M0 (Mann Whitney test: * p < 0.05; ** p < 0.01); (#) points out significant differences between the indicated groups (Mann Whitney test: ## p < 0.01).
(PDF)

S3 Fig Pro-inflammatory cytokine secretion after NLRP3 inflammasome activation in polarized monocytes. Production of IL-1ra and TNF-α cytokines as assessed by ELISA in cell culture supernatants of M0, M1, or M2 monocytes after activation of NLRP3 inflammasome with 100 ng/ml LPS for the indicated time in the presence or absence of 5mM ATP for the last 30 minutes. M0: cells treated with complete medium (control); M1: cells treated with 100 ng/ml IFN-γ (polarized towards M1); M2: cells treated with 10 ng/ml IL-4+IL-13 (polarized towards M2). Data represent the mean ± SEM of ≥ 4 independent experiments done in monocytes isolated from buffy coats of ≥ 4 independent donors.
(PDF)

S4 Fig Pro-inflammatory cytokine secretion after NLRP3 inflammasome activation in polarized macrophages. IL-1ra and TNF-α secretion as assessed by ELISA in cell culture supernatants of M0, M1, or M2 monocytes after activation of NLRP3 inflammasome with 100 ng/ml LPS for the indicated period of times in the presence or absence of 5mM ATP for the last 30 minutes. M0: cells treated with complete medium (control); M1: cells treated with 100 ng/ml IFN-γ (polarized towards M1); M2: cells treated with 10 ng/ml IL-4+IL-13 (polarized towards M2). Data represent the mean ± SEM of ≥ 4 independent experiments done in macrophages derived from monocytes of ≥ 4 independent donors.
(PDF)

S1 Table Primer sequences for qPCR. (PDF)

## Abbreviations

ASC: Apoptosis-associated speck-like protein containing a caspase recruitment domain
DAMP: Damage-associated molecular pattern
IL: Interleukin
LPS: Lipopolysaccharide
LRR: Leucine rich repeat
NLR: Nucleotide-binding domain and leucine-rich repeat containing proteins
NLRP3: Nucleotide-binding, LRR and PYD domains-containing protein 3
PAMP: Pathogen-associated molecular pattern
PBMC: Peripheral blood mononuclear cell
PYD: Pyrin domain
