# Supplementary figures and images for: Impact of human monocyte and macrophage polarization on NLR expression and NLRP3 inflammasome activation
Source: PLoS One. 2017 Apr 12;12(4):e0175336. doi: 10.1371/journal.pone.0175336 (PMC5389804; doi:10.1371/journal.pone.0175336)

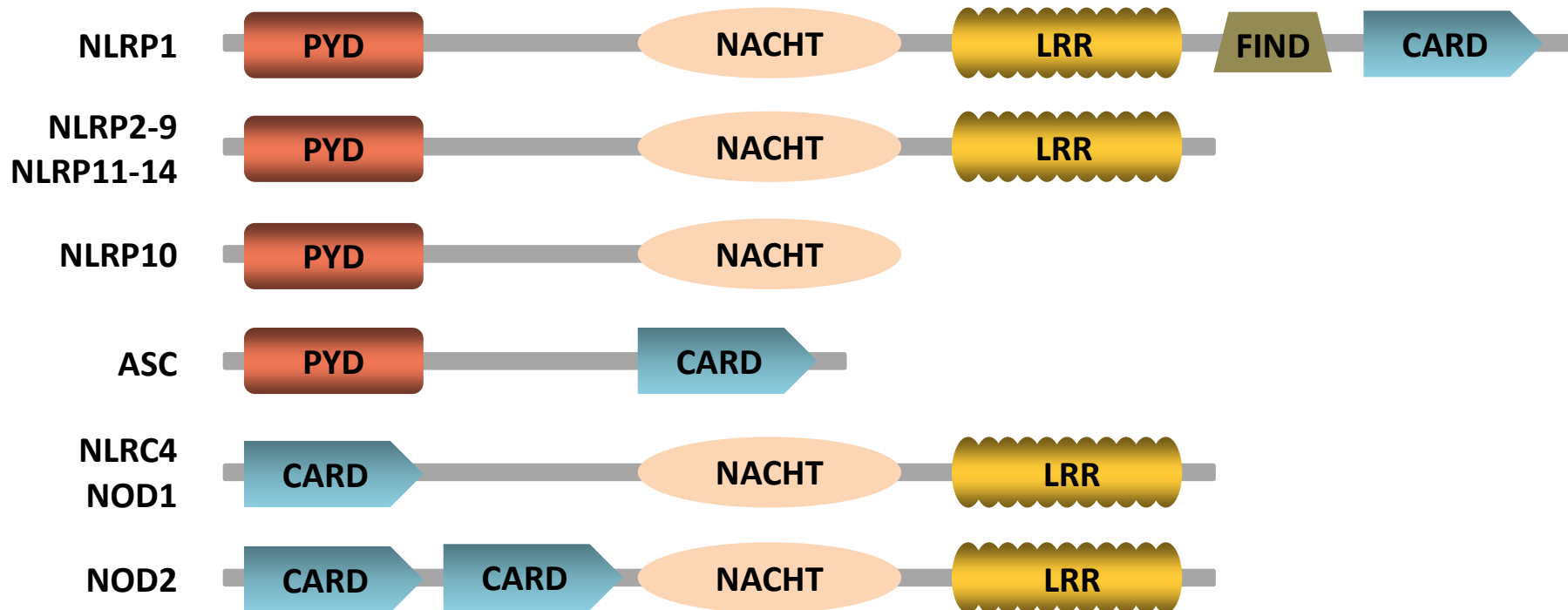

Supplement: S1 Fig — (PDF) [file pone.0175336.s001.pdf]

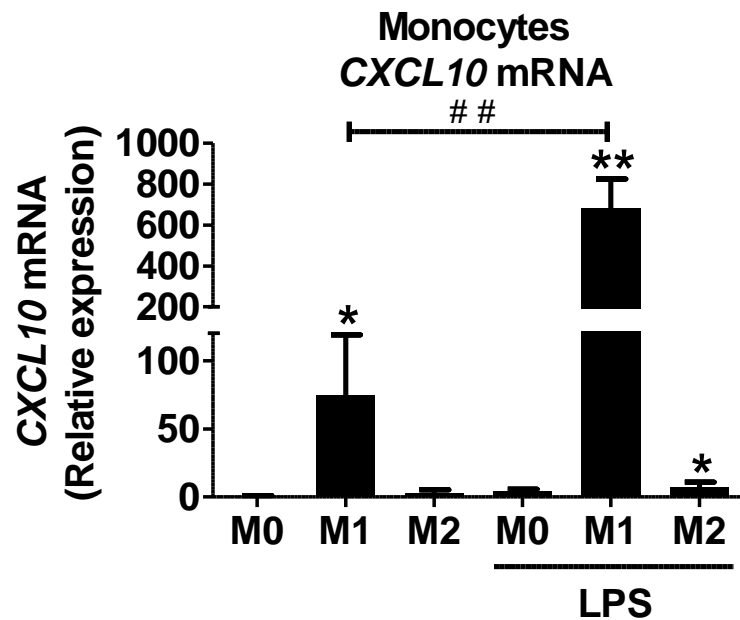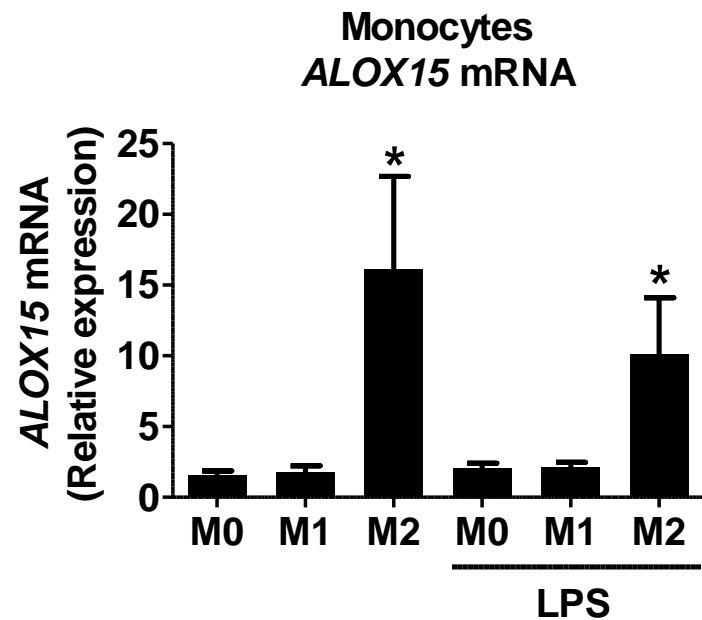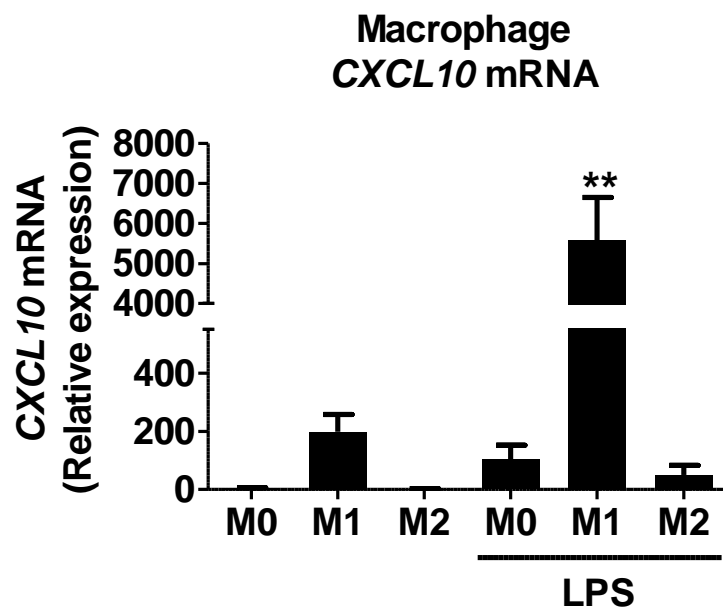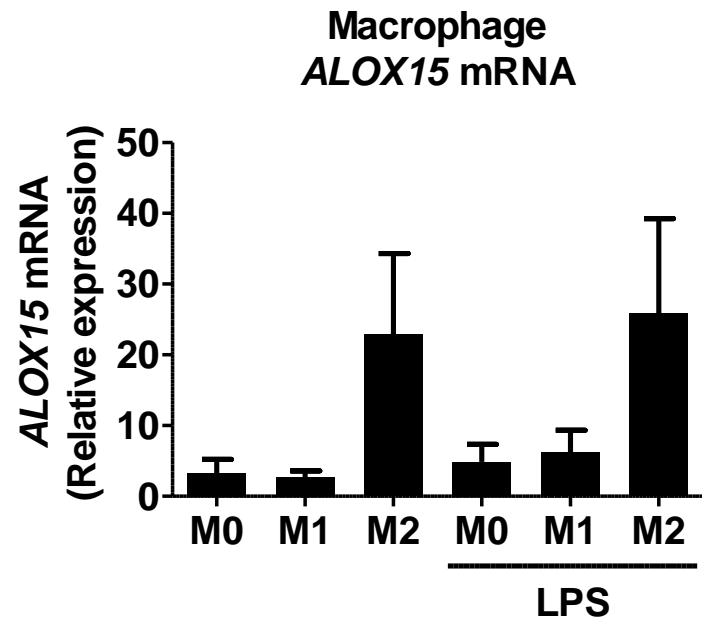

Supplement: S2 Fig — Monocytes and macrophages were polarized towards M1, M2, and stimulated with 100 ng/ml LPS for 3h as described in the methods. mRNA was isolated and gene expression was measured by RT-qPCR and expressed as relative fold change of M0. Data represent the mean ± SEM of ≥ 4 experiments performed in duplicates in cells isolated from ≥ 4 independent donors. M0: cells treated with complete medium (control); M1: cells treated with IFN-γ (polarized towards M1); M2: cells treated with IL-4+IL-13 (polarized towards M2). Asterisks indicate significant differences as compared to M0 (Mann Whitney test: * p < 0.05; ** p < 0.01); (#) points out significant differences between the indicated groups (Mann Whitney test: ## p < 0.01). (PDF) [file pone.0175336.s002.pdf]

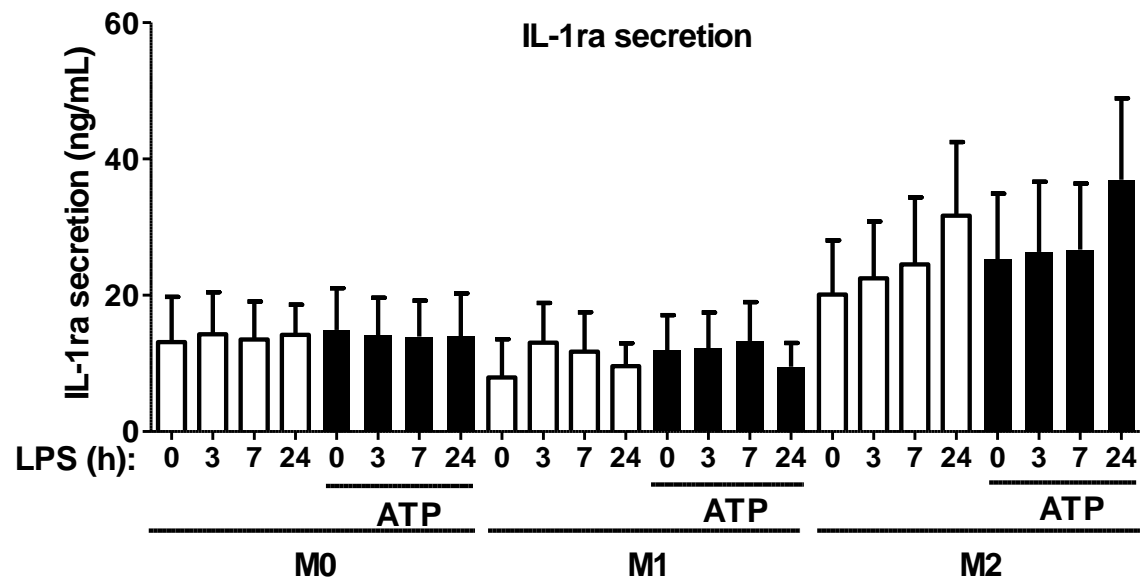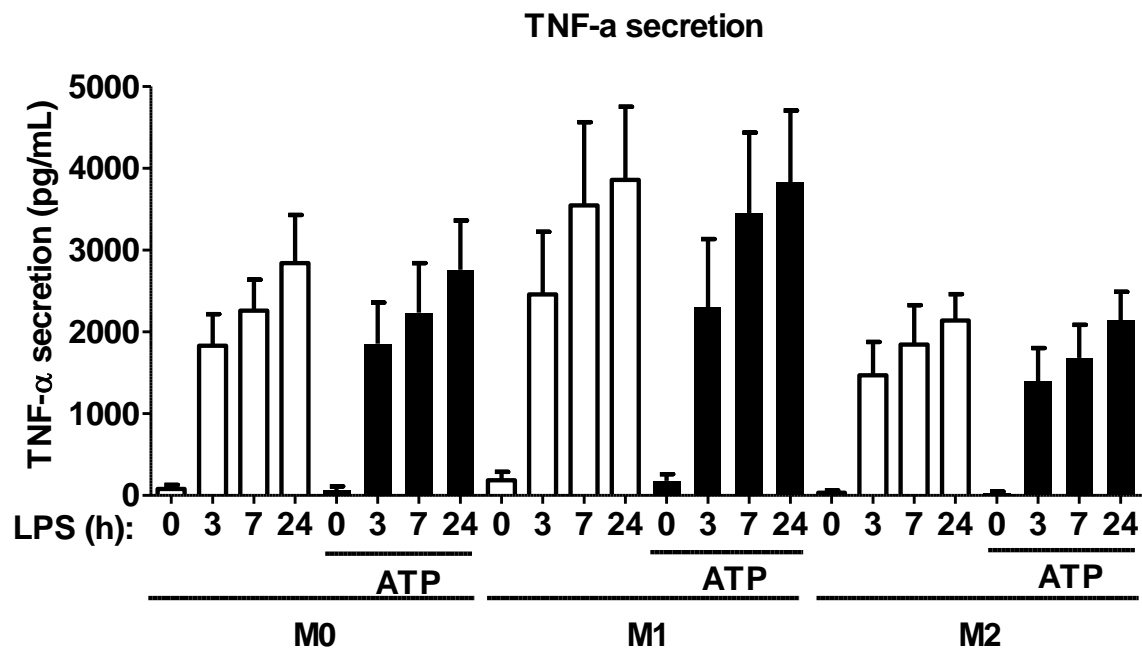

Supplement: S3 Fig — Production of IL-1ra and TNF-α cytokines as assessed by ELISA in cell culture supernatants of M0, M1, or M2 monocytes after activation of NLRP3 inflammasome with 100 ng/ml LPS for the indicated time in the presence or absence of 5mM ATP for the last 30 minutes. M0: cells treated with complete medium (control); M1: cells treated with 100 ng/ml IFN-γ (polarized towards M1); M2: cells treated with 10 ng/ml IL-4+IL-13 (polarized towards M2). Data represent the mean ± SEM of ≥ 4 independent experiments done in monocytes isolated from buffy coats of ≥ 4 independent donors. (PDF) [file pone.0175336.s003.pdf]

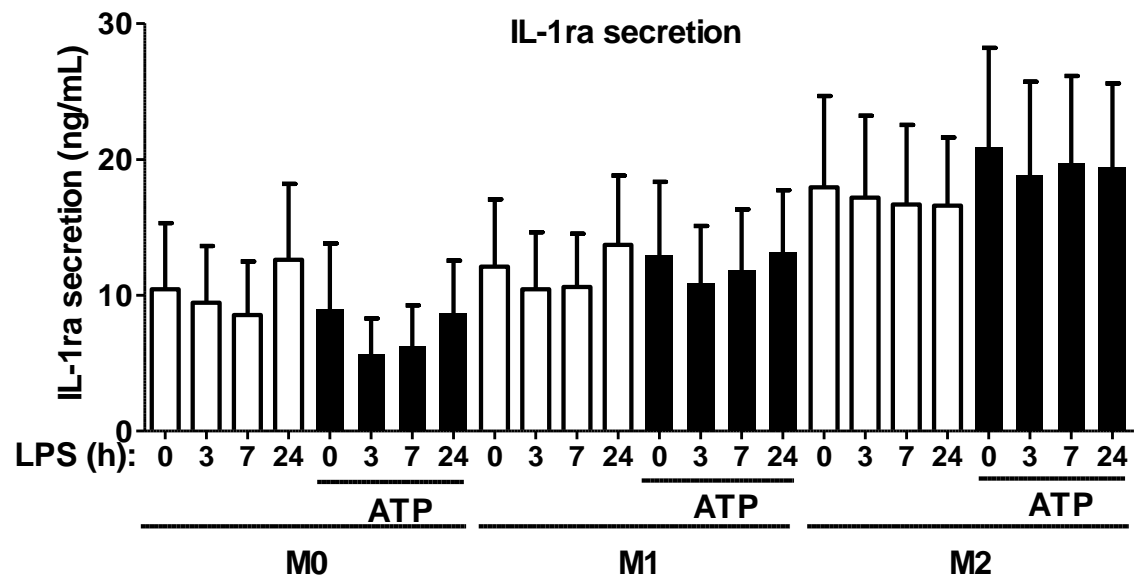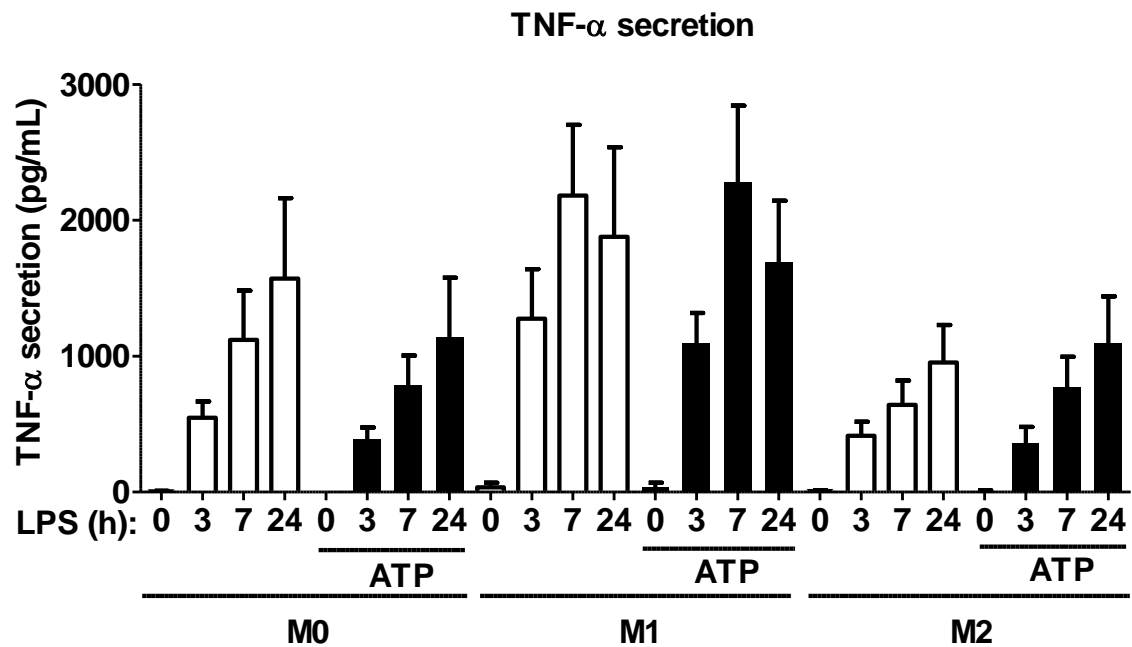

Supplement: S4 Fig — IL-1ra and TNF-α secretion as assessed by ELISA in cell culture supernatants of M0, M1, or M2 monocytes after activation of NLRP3 inflammasome with 100 ng/ml LPS for the indicated period of times in the presence or absence of 5mM ATP for the last 30 minutes. M0: cells treated with complete medium (control); M1: cells treated with 100 ng/ml IFN-γ (polarized towards M1); M2: cells treated with 10 ng/ml IL-4+IL-13 (polarized towards M2). Data represent the mean ± SEM of ≥ 4 independent experiments done in macrophages derived from monocytes of ≥ 4 independent donors. (PDF) [file pone.0175336.s004.pdf]
