# Supplementary material for: Impact of human monocyte and macrophage polarization on NLR expression and NLRP3 inflammasome activation
Source: PLoS One. 2017 Apr 12;12(4):e0175336. doi: 10.1371/journal.pone.0175336 (PMC5389804; doi:10.1371/journal.pone.0175336)
Supplement: S1 Table — (PDF) [file pone.0175336.s005.pdf]

Genes name	Sequences of forward primers	Sequences of reverse primers
RPL13A	CCTGGAGGAGAAGAGGAAAGAGA	TTGAGGACCTCTGTGTATTTGTCAA
MRC1	ACACCAAAACCTGAGCCAAC	CCACCCATCTTCAGTAACTGGT
NLRP1	GGACTGACGATGACTTCTGG	ATCACAAAGCAGAGACCCG
NLRP2	TGTCATCTCCAGAGAGTGGTGT	TTACAGTCTTGTGACCTCGAAGAG
NLRP3	GGAGAGACCTTTATGAGAAAGCAA	GCTGTCTTCCTGGCATATCACA
NLRP6	TCTTCATCCACTCTTTCAGGC	CTCAGAAAGGTCTCGGCAG
NLRP12	GGACTTGAGTTTCAACGACCTG	GTGAGGCCACAGCTATCCAG
NLRC4	CAGTCCCCTCACCATAGAAG	TCAAGTTACCCAAGCTGTCAG
NOD2	CCCTTGAAAGGAATGACACC	CCTCCTCTAGAGAGAAAGTGTTCC
CASP1	CCAGGACATTAAAATAAGGAAACTGT	CCAAAAACCTTTACAGAAGAATCTC
CASP3	TTGTGGAATTGATGCGTGAT	GGCTCAGAAGCACACAAACA
CASP4	TTCCTGGCAATTGAAAATGG	TGCAAGCTGTACTAATGAAGGTG
ASC	TGACGGATGAGCAGTACCAG	GCTTCCGCATCTTGCTTGG
IL1A	GGTTGAGTTTAAGCCAATCCA	TGCTGACCTAGGCTTGATGA
IL1B	AGCTGATGGCCCTAAACAGA	TCGGAGATTCGTAGCTGGAT
IL6	GCCCAGCTATGAACTCCTTCT	CTTCTCCTGGGGGTACTGG
IL18	AACAAACTATTTGTCGCAGGAAT	TGCCACAAAGTTGATGCAAT
TNF	CAGCCTCTTCTCCTTCCTGAT	GCCAGAGGGCTGATTAGAGA
CXCL10	GAAAGCAGTTAGCAAGGAAAGGT	GACATATACTCCATGTAGGGAAGTGA
ALOX15	AGCCTGATGGGAAACTCTTG	AGGTGGTGGGGATCCTGT
